# Isolation of Virulent *Leptospira* Serogroup Australis Field Strains from Symptomatic Dogs for Canine Leptospiral Vaccine Development

**DOI:** 10.3390/microorganisms12101946

**Published:** 2024-09-25

**Authors:** Pierre Bergamo, Marine Le Guyader, Marine Hugonnard, Pascale Bourhy, Nathalie Simon-Dufay, Jérôme Bouvet, Jean-Christophe Thibault, Lionel Cupillard

**Affiliations:** 1 Boehringer Ingelheim Animal Health, Clinical R&D, 01150 Saint-Vulbas, France; jerome.bouvet@boehringer-ingelheim.com; 2 VetAgro Sup, Pôle d’Analyses, Université de Lyon, 69280 Marcy-l’Étoile, France; marine.le-guyader@vetagro-sup.fr; 3 VetAgro Sup, Centre Hospitalier Vétérinaire, 69280 Marcy-l’Étoile, France; marine.hugonnard@vetagro-sup.fr; 4 Unité de Biologie des Spirochètes, Institut Pasteur, National Reference Center and WHO Collaborating Center for Leptospirosis, 75015 Paris, France; pascale.bourhy@pasteur.fr; 5 Boehringer Ingelheim Animal Health, Global Innovation, 813 Cours du Troisième Millénaire, 69800 Saint-Priest, France; nathalie.dufay-simon@boehringer-ingelheim.com (N.S.-D.); lionel.cupillard@boehringer-ingelheim.com (L.C.); 6 JC&P Life Science Partner, 4460 Ancienne Route de Dauphin, 04300 Forcalquier, France; dr.jcthibault@gmail.com

**Keywords:** leptospirosis, Australis serogroup, MAT, molecular diagnosis, bacterial isolation, vaccine

## Abstract

Leptospirosis is a widespread zoonosis caused by spirochaetes belonging to the pathogenic species of *Leptospira*, which are classified into more than 25 serogroups and 250 serovars. Vaccination can prevent the disease in dogs but offers incomplete efficacy because of a lack of cross-protection between serogroups. The aim of this study was to validate a robust recruitment and sampling process, with the objectives of isolating and typing circulating *Leptospira* pathogenic strains and then selecting those of proven virulence and pathogenicity for vaccine development. Blood and urine samples from dogs with clinical syndromes compatible with acute leptospirosis were sterilely collected and transported to a reference laboratory for a micro-agglutination test (MAT), PCR, and bacterial isolation. Isolated strains underwent molecular typing using RNA16S, variable-number tandem repeat (VNTR), and pulsed-field gel electrophoresis (PFGE). Subtyping was performed using core genome multilocus sequence typing (CgMLST). Among 64 included dogs, 41 had MAT and/or PCR results compatible with *Leptospira* infection, and 14 *Leptospira* strains were isolated. Based on molecular typing, 11 isolates were classified as *L. interrogans* serogroup Australis, serovar Bratislava, and 3 as serogroup Icterohaemorrhagiae, serovar Icterohaemorrhagiae. CgMLST subtyping revealed a diversity of clonal groups (CGs) distributed in several regional clusters. Besides validating a robust recruitment and sampling process, this study outlines the value of combining PCR and serological testing when suspecting leptospirosis and the usefulness of implementing molecular typing methods to identify circulating field strains. It also confirms the epidemiological importance of the Australis serogroup and allows for the collection of different highly pathogenic strains for vaccine development.

## 1. Introduction

Leptospirosis is a widespread zoonosis caused by spirochaetes belonging to the pathogenic species of the genus *Leptospira*. Estimates indicate that the annual incidence in humans exceeds one million cases worldwide [1,2,3]. Clinical signs in dogs range from subclinical to severe and potentially fatal disease with multi-organ dysfunction [4,5].

Prevention relies on sanitary measures and vaccination. Vaccines offer incomplete efficacy because multiple and non-cross-protecting serogroups are involved in the disease [5]. Consequently, they should include serogroups that are relevant to a certain geographical area. Previously published data based on MAT have revealed the presence of various serogroups in France, among which, besides Icterohaemorrhagiae, and, to a lesser extent, Grippotyphosa, Australis plays an important role [6,7,8]. Consequently, vaccines that include these serovars have been brought to the market over the last decade, in particular, with one of those having recently been introduced.

Laboratory tests are necessary to confirm the diagnosis of clinically suspected leptospirosis due to the non-pathognomonic symptomatology and wide range of symptoms. *Leptospira* can be cultured from blood and urine, but the recovery of isolates from clinical specimens is technically difficult due to the slow growth of this bacterium and its high susceptibility, particularly to contaminating organisms. An additional complexity is the need for dark-field microscopy, which necessitates an experienced operator to assess culture positivity [9,10,11]. Molecular techniques, such as those based on the polymerase chain reaction (PCR), are now more commonly used for diagnostic purposes [11,12,13]. A serological diagnosis relies mainly on the microscopic agglutination test (MAT), still considered today as the reference technique. For the MAT, a minimum of two serum samples should be taken at 2- to 4-week intervals, particularly when the first sample is taken at an early stage of the disease. More recently, a variety of newer serological tests, such as IgM and/or IgG enzyme-linked immunosorbent assays (ELISAs)—both as laboratory and point-of-care tests—have been developed [13,14,15,16].

Even if an MAT performed on two consecutive samples can provide useful information, this technique has known limitations [11,17,18,19,20,21]. One of the definitive ways to determine the infecting serogroups and their epidemiological relevance is to isolate leptospires and/or conduct molecular typing. Different molecular methods have been developed to better identify causative serovars, such as multilocus sequence typing (MLST), variable-number tandem repeat (VNTR), and multilocus variable-number tandem repeat analysis (MLVA). Other methods include mass spectrometry (MALDI-TOF) and a restriction fragment length polymorphism (RFLP) based on pulsed-field gel electrophoresis (PFGE) [13,22,23,24,25]. Recently developed PCR techniques, such as those targeting the rfb gene locus responsible for LPS biosynthesis, are now also able to provide such information and are much easier to implement [26,27].

The objectives of this study were first to validate a robust recruitment and sampling process and then to isolate and type circulating *Leptospira* pathogenic strains in view of selecting strains of proven virulence and pathogenicity for further vaccine development.

## 2. Materials and Methods

### 2.1. Selection of Veterinary Practices

Twenty-two practices that are located in known leptospirosis hotspots and/or regularly reporting clinical cases were selected. The overall duration of the study was 4 years, with the first phase taking place from August 2010 to October 2011. The second phase took place from May 2013 to October 2014 in order to increase the number of cases and extend the geographical area of sample collection.

### 2.2. Animals

Each practice was asked to include dogs for which leptospirosis was suspected based on at least one of the following criteria:

- Acute renal failure associated with general clinical signs of recent and sudden onset (i.e., apathy, lethargy, dehydration), digestive signs (i.e., anorexia, diarrhea, vomiting), and urinary signs (i.e., oligo-anuria, polyuria-polydipsia, increase in kidney size, renal pain).
- Glucosuria without hyperglycemia.
- Acute hepatitis associated with general clinical signs of recent and sudden onset (i.e., icterus, apathy, lethargy, dehydration) and digestive signs (i.e., anorexia, diarrhea, vomiting).
- (Non-parvoviral) acute hemorrhagic gastroenteritis.
- Hemorrhagic syndrome.

Dogs who had received antibiotic treatment within the previous 3 weeks were excluded from this study.

Dog owners were asked to fill in an informed owner consent form.

### 2.3. Sample Collection and Clinical Sign Recording at Inclusion

Seven sets of six pre-identified tubes were provided to each investigator, with each set containing one 4 mL EDTA tube (“Whole blood, D0”) for PCR purposes, two 4 mL plain tubes (“Serum D0” and “Serum D 14–21”) for serological testing, one 11 mL tube containing PBS (“Urine PBS D0”), and two 11 mL tubes filled with 9 mL Ellinghausen–McCullough–Johnson–Harris (EMJH) medium (“Urine EMJH, D0” and “Blood EMJH, D0”) for bacterial isolation. In addition, the investigators were provided with 10 non-identified dry tubes with a clotting activator.

On the day of inclusion (D0), dogs underwent a clinical examination, and clinical signs were recorded.

Then, 3 mL of urine was collected via cystocentesis in a sterile manner for *Leptospira* isolation and PCR testing; 2 mL was immediately transferred to the corresponding pre-identified PBS tube and 5 to 10 drops were added to the pre-identified EMJH tube for the primary bacterial culture. The tubes were stored at room temperature until shipment.

On the same day, about 6–7 mL of blood was collected. Five to ten drops were transferred to the corresponding pre-identified EMJH tube, whereas 3 mL was transferred to the pre-identified dry EDTA tube and 3 mL was transferred to a non-identified 4 mL dry tube with a clotting activator. In this latter tube, the blood was allowed to decant for one hour at room temperature and was then centrifuged for 10 min. The supernatant was then transferred to the corresponding serum pre-identified dry tube. The tubes were stored at room temperature until shipping.

**Figure 1:**
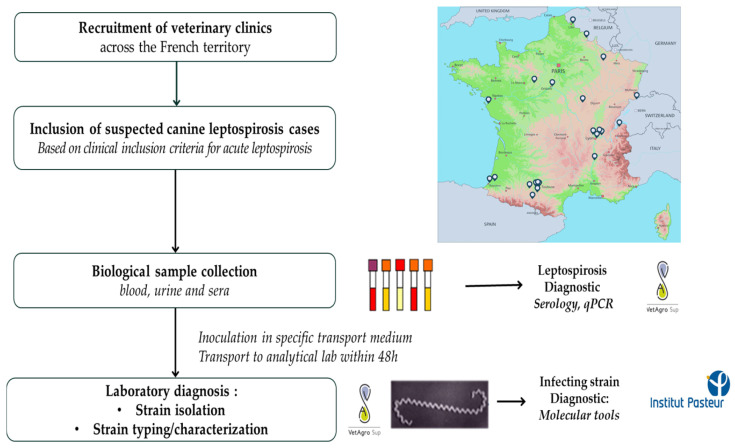
Summary of the study design.

If possible, a second 3 mL sample of blood was taken between days 14 and 21 and processed for serum recovery as previously described (Figure 1).

### 2.4. Shipment

To maximize the chance of isolating and identifying causative *Leptospira* strains, special care was taken with the transportation of the samples. A dedicated courier service was organized at the veterinary practice level, ensuring that the samples reached the reference laboratory at VetAgroSup, Marcy l’Etoile, as quickly as possible, i.e., within 48 h of the samples being collected at the latest.

### 2.5. Laboratory Analysis

#### 2.5.1. Serological Testing

The MAT was performed at the Laboratoire des Leptospires (VetAgro Sup, Marcy l’Etoile), according to the WOAH guidelines [28]. Sera were tested against 20 pathogenic or intermediate leptospiral serovars belonging to 10 serogroups (Table 1). The endpoint titers were determined using serial two-fold dilutions from 1/40 to 1/20,480 (first study phase) and from 1/100 to 1/12,800 (second study phase). After 2 h of incubation at 37 °C, Leptospires agglutinations were observed via dark-field microscopy, and the last well showing 50% agglutination was recorded. A 1/40 dilution was the first dilution step during the first phase of this study, and a 1/100 dilution was the first dilution step during the second phase of this study.

#### 2.5.2. Leptospira Detection

DNA was extracted using a KingFisher^®^ instrument (Thermo Scientific, Illkirch, France) and a universal kit (LSI MagVet Universal Kit MV384, Thermofisher, Courtaboeuf, France) using 200 μL of blood or a pellet from 1 mL of centrifuged urine (3000× *g* for 10 min at room temperature). When the blood or urine quantity was insufficient for a separate analysis (one case), the blood and urine were pooled before the extraction protocol. The presence of *Leptospira* DNA was assessed using a specific pathogenic *Leptospira* TaqMan real-time PCR kit (TaqVet PathoLept, Life technologies, Villebon sur Yvette, France). qPCR was performed according to the manufacturer’s instructions, using 20 µL of commercial PCR mix and 5 μL of extracted DNA. A positive control with Icterohaemorrhagiae DNA and a negative control with nuclease-free water were included by replacing the sample DNA. The absence of PCR inhibitors in the samples was controlled using an internal control amplification system. Samples with a cycle threshold (Ct) of less than 45 cycles were considered positive, in accordance with the manufacturer’s instructions.

#### 2.5.3. Bacterial Isolation

In order to eliminate possible contaminating bacteria, the 1/10 dilution primary culture was further diluted to 1/100 and then to 1/1000 in a 30 °C pre-heated EMJH medium, according to the standard operating procedures of the Laboratoire des Leptospires (VetAgro Sup, Marcy l’Etoile, France).

All 3 blood inoculated tubes and 3 urine inoculated tubes were then cultured at 30 °C +/−2 °C for 3 months. Bacterial growth was observed each week via dark-field microscopy. When obtaining a pure and dense culture (minimum 1.10^7^ bacteria/mL), 1 mL of the culture was deep-frozen in a cryotube in liquid nitrogen with 5% DMSO until further processing.

#### 2.5.4. Leptospira Genotyping and Assignation to Serogroup and Serovar

Molecular characterization was carried out using *Leptospira* DNA extracts from positive urine or blood samples (when there was sufficient DNA) and confirmed from the isolated strain.

- PCR 16S

DNA was extracted from the isolated strain using a QIAamp DNA Mini Kit (Qiagen, Courtaboeuf, France) following the manufacturer’s recommendations, and a conventional PCR targeting the partial 16S gene, encoding the subunit of the 16S ribosomal RNA, was performed according to the techniques described by Ayral [29], Mérien [30], and Le Guyader [24].

PCR products were sequenced by Genoscreen (Lille, France). Contigs were obtained with ChromasPro software (v. 2.6.6), and species were assigned by sequence alignments using NCBI nucleotide BLAST (http://blast.ncbi.nlm.nih.gov, accessed on 27 March 2024).

- VNTR

A variable-number tandem repeat (VNTR) analysis was carried out at the Laboratoire des Leptospires (VetAgro Sup, Marcy l’Etoile) with the amplification of the VNTR4, VNTR7, and VNTR10 loci according to Salaün and to Le Guyader [22,24]. The VNTR profile identified for each locus was used to deduce the *Leptospira* genotype and putative serovar assignation in relation to the published database [22].

- CgMSLT

Core genome MLST (cgMLST) was performed at Institut Pasteur, Paris, using a scheme based on 545 core genes, as previously described [31]. Briefly, Illumina sequencing was performed using the extracted genomic DNA of exponential-phase cultures using a MagNA Pure 96 Instrument (Roche, Meylan, France). Next-generation sequencing (NGS) was performed using a Nextera XT DNA Library Preparation kit and NextSeq 500 sequencing systems (Illumina, San Diego, CA, USA) at the Mutualized Platform for Microbiology (P2M). CLC Genomics Workbench 9 software (Qiagen, Hilden, Germany) was used for analyses. The generated contig sequences were downloaded in BIGSdb hosted at the Institut Pasteur (https://bigsdb.pasteur.fr/leptospira/, accessed on 27 March 2024). The genomes were then compared with available genomes in our database, including those of reference strains. A clonal group (CG) is defined as a group of cgMLST allelic profiles differing by no more than 40 allelic mismatches out of 545 gene loci [31,32,33,34]. cgMLST was used to deduce a putative serogroup for the tested strains.

- PFGE

Pulsed-field gel electrophoresis (PFGE) was also performed at Institut Pasteur. The cells of an exponential-phase culture were embedded in agarose plugs as previously described [32,33,34]. DNA plugs were restriction digested with NotI. PFGE was performed in a contour-clamped homogeneous electric field DRIII apparatus (Bio-Rad Laboratories, Richmond, CA, USA). Programs with ramping from 1 to 70 s for 36 h at 150 V and from 10 to 100 s for 40 h at 150 V were used to resolve restriction patterns. PFGE profiles were compared with those of the reference strains. If the PFGE profile of the strain of interest is indistinguishable or closely related (less than three band differences) to that of a reference serovar, the strain is considered to belong to that serovar [2,3,4].

### 2.6. Determination of Infection Status Based on MAT and PCR Results

*Leptospirosis* was considered to be confirmed when at least one of the following conditions was fulfilled:

(1) Serology (MAT) was consistent with a leptospiral infection: one or more titers ≥640 (first study phase) or ≥800 (second study phase) for non-vaccinal serogroups on at least one of the serum samples (MAT+).
(2) A positive PCR result was obtained for at least one sample (urine, blood).
(3) A sample was positive for bacterial isolation.

Dogs negative for all serogroups in the MAT, and dogs positive for vaccinal serogroups only and negative for non-vaccinal serogroups (vaccinal serological profile), were considered as not having a serological profile consistent with infection (MAT−).

Dogs displaying high titers for a vaccinal serogroup (s) (Li and/or Lc, i.e., >640 or 800 depending on the study phase), associated with low titers to non-vaccinal serogroups (i.e., <640 or 800 depending on the study phase), were classified as doubtful (MAT±).

## 3. Results

### 3.1. Overall Population Characteristics

Sixty-four suspected acute leptospirosis cases were included over a 3.5-year period.

#### 3.1.1. Sex and Age

- A total of 16 dogs were intact females, 12 were neutered females, 32 were intact males, and 4 were neutered males.
- The recruited dogs were 8.0 years old on average [0.2–15.6]: 7 dogs were below 2 years of age, 9 were between 2 and 5 years of age, 25 were between 5 and 10 years of age, and 21 were older than 10 years. No data were available for two dogs.

#### 3.1.2. Vaccinal Status

All dogs, if vaccinated, had received a bivalent vaccine containing the Icterohaemorrhagiae and Canicola serogroups.

- Fifteen dogs had a correct vaccination background against leptospirosis (as defined by the vaccine marketing authorization).
- Thirty dogs had an incorrect vaccination status (mainly overdue vaccination), and seventeen dogs were not vaccinated at all. Data were missing for two dogs.

#### 3.1.3. Habitat

- A total of 15 dogs lived exclusively outdoors, 34 lived indoors/outdoors, and 15 lived indoors with limited outdoor access.
- A total of 70% of the dogs (45/64) had regular access to natural, open waters; 69% (31/45) had access to swampy/standing waters; and 35% (16/45) had access to ponds/lakes, whereas 5 had exclusive access to running waters (rivers and springs). Sixteen dogs had access to drinking troughs, and thirteen had no access at all to natural, open waters.
- A total of 18 dogs were hunting dogs, and close to half of all dogs (31/64) lived in multidog households.
- In addition, 49 out of the 64 included dogs were declared as having close contact with rodents.

#### 3.1.4. Clinical Presentation at Inclusion

Forty-two (65.6%) dogs presenting an acute renal failure syndrome (ARF) were recruited (Table 2):

- Eighteen (28.1%) presented this syndrome as the sole criterion.
- Eighteen (28.1%) presented an association with one other syndrome: nine (14%) with glucosuria without hyperglycemia (GluU), four (6.3%) with acute hepatitis (AH), four (6.3%) with acute hemorrhagic gastroenteritis (AHGE), and one (1.6%) with a hemorrhagic syndrome (HS).
- Six (9.4%) presented a combination of three syndromes: three (4.7%) presented ARF + AH + GluU, and three (4.7%) presented ARF + AH + AHGE.
- Fifteen dogs (23.4%) were recruited based on an AH syndrome without ARF:
- Twelve (18.8%) with AH as the sole criterion.
- Three (4.7%) with AH + AHGE.
- Six dogs (9.4%) were primarily included based on AHGE without ARF or AH:
- Five (7.8%) as the sole criterion.
- One (1.6%) in association with an HS.

Finally, one dog (1.6%) was included based on “glucosuria without hyperglycemia” only.

**Table 2:** Clinical syndromes observed at inclusion in the 64 recruited dogs.

With ARF at inclusion	ARF Alone	18				18
	+AH	4	Without ARF at inclusion	AH	12	16
	+AHGE	4		AHGE	5	9
	+HS	1		Hs	0	1
	+GluU	9		GluU	1	10
	+AH + AHGE	3		AH + AHGE	3	6
	+AH + GluU	3		AH + GluU	0	3
	+AH + HS	0		AHGE + Hs	1	1
	Total	42			22	64

ARF, acute renal failure; AH, acute hepatitis; AHGE, acute hemorrhagic gastroenteritis; HS, hemorrhagic syndrome; GluU, glucosuria without hyperglycemia.

Of the 64 dogs, 21 (32.8%) died within the observation period (D0-D21). Death occurred on average 3.1 days after inclusion [0–10] and 5.4 days [1–18] after the reported onset of clinical signs. In addition, 2 dogs died outside the observation period: one dog at 47 days and the other at 50 days after inclusion.

In summary, two-thirds of the dogs (42/64, 65.6%) were included based on the occurrence of acute renal failure syndrome (ARF), among which 42.9% (18/42) solely expressed this syndrome. ARF was observed in combination with other syndromes in 24 out of 42 dogs (57.1%), mostly with one other syndrome (42.9% of cases (18/42)) or in a multi-syndromic setting (ARF + AH and/or GluU and/or AHGE) (14.3% of cases (6/42)).

In the absence of ARF, other syndromes leading to inclusion were AH (15/64, 23.4%) and AHGE (6/64 = 9.4%), associated or not associated with another syndrome. GluU alone led to the inclusion of one dog.

### 3.2. Laboratory Results (MAT and PCR)

A first set of blood and urine samples was obtained from all 64 dogs on the day of inclusion. Among these 64 cases, blood samples could be taken again from 28 (43.8%) dogs after the first sampling. A second sample of blood could not be drawn from 36 dogs (56.2%), 21 because they died within the observation period, and 15 because they did not show up again. 

Amongst all the included dogs, 34 (53.1%) had a serological status compatible with an active leptospiral infection (MAT+); 23 (35.9%) had a negative status, meaning that serology was not compatible with an active *Leptospira* infection (MAT−); and 7 (10.9%) were doubtful (MAT±). A total of 21 (32.8%) dogs were PCR-positive: 6 (9.4%) solely on blood, 11 (17.2%) solely on urine, 3 (4.7%) on both blood and urine, and 1 (1.6%) on a “blood + urine” pool.

Taking into account both diagnostic tests, leptospiral infection was confirmed in 41 out of the 64 cases (64%), either via the MAT and/or PCR: 14 dogs were MAT+ and PCR+, 20 were MAT+ and PCR−, 5 were MAT−/PCR+, and 2 were MAT±/PCR+. Finally, 18 dogs remained both PCR- and MAT-negative, and 5 dogs were PCR−/MAT± (Table 3). Among the 23 death cases, 10 dogs were both MAT- and PCR-positive, 8 were MAT+ and PCR−, 1 was MAT±/PCR−, and 4 were both PCR- and MAT-negative.

Among the 23 death cases, 10 dogs were both MAT- and PCR-positive, 8 were MAT+ and PCR−, 1 was MAT±/PCR−, and 4 were both PCR- and MAT-negative.

### 3.3. Clinical Presentation of the 41 Infected Dogs

Acute renal failure was the main syndrome at inclusion in 32 (78.0%) of the 41 *Leptospira*-infected dogs, whereas this was the case for 7 out of the 18 dogs (38.9%) with a negative leptospiral status. Glucosuria w/o hyperglycemia was the second most common syndrome at inclusion, presenting in 10 dogs out of 41 (24.4%), whereas this was the case for only 1 dog with a negative leptospiral status (Table 4).

ARF was the sole syndrome in 14 (34.1%) of all the 41 infected dogs. ARF was associated with the following other syndromes in 18 cases (43.9%): AH (7 cases), and/or AHGE (5 cases), and/or GluU (10 cases). ARF was associated with an HS in only one case. AH without ARF was present in eight cases; it was present as the sole syndrome in five cases and associated with AHGE in three cases. When GluU was one of the inclusion criteria, it was always associated with ARF, either alone (seven cases) or together with AH (three cases). AHGE was seen as the sole syndrome in only one case, and HS and GluU were never seen as the sole syndrome (Table 5).

Detailed clinical data at inclusion were available for 39 out of the 41 infected dogs. The most common general clinical signs in these dogs were lethargy in 94.9% (37/39), anorexia in 87.2% (34/39), dehydration in 48.7% (19/39), abdominal pain in 38.5% (15/39), icterus in 28.2% (11/39), weight loss in 28.2% (11/39), and muscular pain/weakness in 23.1% (9/39). Digestive signs were also frequently reported, with vomiting in 64.1% (25/39), whereas diarrhea, frequently with hematochezia and/or melena, was observed in 35.9% (14/39) of the cases. Urinary signs were inconsistently observed, either as oligoanuria in 15.4% (6/39) or polyuria/polydipsia (PUPD) in 20.5% (8/39) of the dogs. Four dogs (10.3%) displayed ocular congestion. Signs of kidney damage (hypertrophy and/or pain) and/or hepatomegaly were present in 15.4% (6/39) and 7.7% (3/39) of the cases, respectively. Other clinical signs, such as hemorrhagic, neurological, or respiratory signs, were less commonly reported, usually in less than 7% of the cases.

Rectal temperature abnormalities were rather inconsistent, with a similar proportion of dogs showing hyperthermia (15.9%) and hypothermia (15.9%) and 68% being normothermic. No data were available for two dogs (Table 6).

### 3.4. Bacterial Isolation

*Leptospira* strains were isolated from 14 dogs: strains were isolated from blood only in 7 dogs, whereas in 6 dogs, strains were isolated from urine only; in 1 dog, a strain was recovered both from blood and urine.

From a clinical point of view, ARF was present at inclusion for all these dogs, whereas only four displayed AH, and only two displayed AHGE. GluU was present in five dogs.

### 3.5. Consistency between PCR and Bacterial Isolation

A positive culture was fully consistent with the PCR results (“blood culture +/blood PCR +” or “urine culture +/urine PCR +”) in five of the clinical cases (A02, A07, A09, Q02, and T01). In four other cases, the culture was partially consistent with the PCR results, with either the PCR being positive for both blood and urine (or in a pooled sample for case F01, see Supplementary Materials) but the culture being positive only for the blood sample (B02, B06, and F01) or the PCR being positive only for urine but the culture being positive for both blood and urine samples (S03). In three cases, some discrepancy was seen, with the PCR being positive for urine but the culture being positive for blood (H03 and Q01) or the PCR being positive for blood but *Leptospira* being isolated from urine only (U01). Finally, *Leptospira* was isolated from either blood or urine in two cases where the PCR was negative for both blood and urine (B01 and H01) (Table 7).

### 3.6. Strain Characterization

#### 3.6.1. Serological Characterization Using MAT

Based on the MAT results (BS1 and/or BS2 when available), the Australis serogroup was considered as being “very likely” or “likely” involved in 48.8% (20/41) of the positive cases, whereas 5 cases were likely related to the Icterohaemorrhagiae serogroup; 16 cases remained inconclusive (Supplementary Materials Table S1).

Focusing more specifically on the fourteen cases with bacterial isolation and based on the highest observed MAT titers, infection was very likely to be related to the Australis serogroup in six cases and to the Icterohaemorrhagiae serogroup in three cases. The interpretation was doubtful in four cases, with three dogs likely being infected by Australis and one likely being infected by Icterohaemorrhagiae. One case with only one blood sample (B01) was inconclusive (Table 8).

#### 3.6.2. Molecular Typing

Based on RNA16S sequencing, all the 14 isolated strains were identified as belonging to the pathogenic *L. interrogans* species. Following molecular typing, nine strains were confirmed as belonging to the Australis serogroup, and three strains were confirmed as belonging to the Icterohaemorrhagiae serogroup.

**Figure 2:**
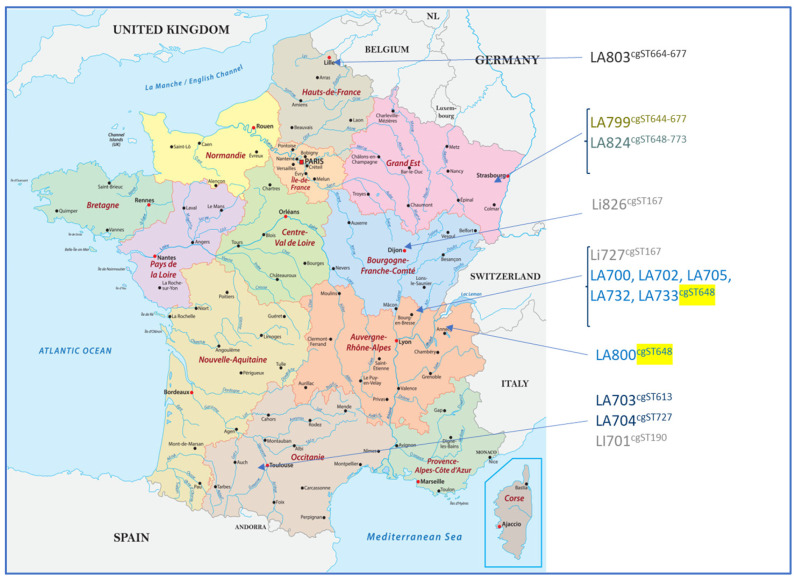
Geographical distribution of the isolated strains with their corresponding cgMLST profiles. Six strains from the same geographical region (Rhône-Alpes) displayed the same profile (cgST648, as highlighted in yellow), possibly reflecting the existence of a geographical cluster. The other *Leptospira* Australis serogroup isolates presented various cgST profiles.

High-resolution typing using cgMLST revealed a high diversity among the isolates regardless of the serogroup. cgMLST allowed for the determination of two clonal groups (CGs): one corresponding to strains belonging to the *L. interrogans* serogroup Icterohaemorrhagiae (CG6) and the other corresponding to the *L. interrogans* serogroup Australis (CG69). Within each CG, strains sharing the same sequence type (cgST) could be identified. CG6 contained two strains both located in Central–East France with the same cgST (cgST167) and another isolated in South-West France with a unique cgST (cgST190). For CG69, six cgSTs could be found among the 11 strains, with 6 strains from the same geographical region (Rhône-Alpes) displaying the same profile (cgST648), possibly reflecting the existence of a geographical cluster (Table 9 and Figure 2).

#### 3.6.3. Consistency between Serological Characterization When Using MAT and Molecular Typing on Isolates

A total of 7 out of the 14 cases with bacterial isolation that were initially “undoubtfully” assigned to a serogroup based on the MAT were confirmed as such via molecular typing: 6 belonged to serogroup Australis (A02, H01, H03, Q01, Q02, and T01) and 1 belonged to serogroup Icterohaemorrhagiae (F01). In addition, three unclear MAT results (as indicated in Table 8 and Table 10) were also confirmed via molecular typing: two as Australis (A09 and U01) and one as Icterohaemorrhagiae (S03). The one inconclusive MAT case (dog B01) was identified as serogroup Australis via molecular typing. One case classified as likely being infected by serogroup Australis was actually infected by Icterohaemorrhagiae (A09). Finally, two cases initially classified as being infected by Icterohaemorrhagiae based on the MAT results were not confirmed as such via molecular typing and were actually infected by a *Leptospira* serogroup Australis strain (B02 and B06) (Table 10).

#### 3.6.4. Clinical Presentation of the 14 Isolation-Positive Dogs at Inclusion

All 14 dogs presented ARF at inclusion. In addition, four of them (two serogroup Australis- and two serogroup Icterohaemorrhagiae-infected dogs) also displayed AH, and five displayed GluU. AHGE was present in only two dogs, both infected by Australis (Table 11). All 11 serogroup Australis-infected dogs exhibited lethargy and anorexia. Five of these latter dogs (45.5%) also exhibited vomiting, and four (36.4%) exhibited signs of abdominal pain. Renal/urinary symptoms were characterized by the presence of oligoanuria in three of the serogroup Australis-infected dogs (27.2%). Icterus was observed in only one of the serogroup Australis-infected dogs but in two of the three serogroup Icterohaemorrhagiae-infected dogs.

Seven out of the eleven serogroup Australis-infected dogs survived acute leptospiral infection, as well as one of the three serogroup Icterohaemorrhagiae-infected dogs.

All cases, whether infected by serogroup Australis or Icterohaemorrhagiae, presented acute renal failure syndrome, whereas acute hepatitis and acute hemorrhagic syndrome were less commonly observed. The overall survival was 57.1%, showing the high pathogenicity of leptospiral infection.

## 4. Discussion

This study was conducted in order to validate a robust recruitment and sampling process, with the aim of isolating and typing circulating pathogenic *Leptospira* strains in view of selecting strains of proven virulence and pathogenicity for vaccine development.

The sample collection and processing implemented during this study proved to be robust, as it allowed for the isolation of 14 *Leptospira* strains from either blood, urine, or both. This represents a success rate of 21.9% when considering all 64 included cases and 34.1% among the 41 dogs considered as infected. These are fairly satisfactory results, taking into account that the samples had to be transported, often over a long distance, to reach the laboratory and considering that *Leptospira* are highly susceptible, difficult-to-grow bacteria, particularly when attempting to culture them from potentially non-sterile sources such as urine [35]. To overcome this issue and to improve the isolation rate, all samples were taken in a sterile manner—in particular, urine was collected via cystocentesis—and were immediately inoculated at the practice level into an EMJH growth medium. Ensuring that the samples reached the laboratory within 48 h contributed to increasing the isolation rate. As already stated in the literature, the most important factors for the successful isolation of *Leptospira* are aseptically collected material, quick processing, a suitable culture medium, and selective antibiotics [36,37,38].

Considering the relevance of case recruitment, leptospirosis is a multi-systemic disease affecting the kidneys and the liver in particular [7,8,13,36,39]. Acute renal failure, acute hepatitis, and glucosuria without hyperglycemia were therefore chosen as major recruitment criteria. Since hemorrhagic syndrome is also associated with leptospiral infection, we added this criterion to the recruitment parameters, as well as non-parvoviral acute hemorrhagic gastroenteritis [5]. By applying these criteria, we were able to succeed in recruiting 41 infected dogs (MAT- and/or PCR-positive) among 64 clinically suspicious cases, which represents a notable success rate of 64%.

Our study showed that acute renal failure and/or glucosuria without hyperglycemia were likely to be fairly strong discriminating factors for *Leptospira* infection, with 78.0% of the positive dogs presenting ARF and 24.4% presenting glucosuria, compared with 38.9% and 5.6% in dogs with a negative leptospirosis status, respectively. There was an even stronger likelihood for a dog to be infected when the two inclusion criteria were observed together, as infection was confirmed in 10/12 dogs (83.3%) presenting this association. In contrast, the presence of acute hepatis and/or acute hemorrhagic gastroenteritis was not a discriminating factor at inclusion.

From a diagnostic point of view, the MAT remains the gold-standard technique at the clinical practice level for confirming *Leptospira* infection. However, the MAT should be interpreted very cautiously at the individual level, particularly in the early stages of the disease and/or in the absence of a second sample taken after 2 to 4 weeks. As a matter of fact, in our study, 24 dogs were considered negative and 7 were considered doubtful based on the MAT performed on the first sample, among which 5 proved to be positive based on PCR and/or isolation. Two dogs (Q01 and Q02) from which *Leptospira* was isolated but who were initially MAT-negative became strongly MAT-positive on the second blood sample. This shows that “negative” cases, for which infection was not demonstrated using the MAT (MAT− or MAT+/−), should not necessarily be considered “non-infected” cases, particularly when there is no second blood sample. This also applies to cases with low MAT titers that might have been considered negative, as a high positivity threshold at 1/640–1/800 was chosen to determine the infected status.

These data confirm that a reliable *Leptospira* diagnosis cannot be obtained based on a single MAT and that PCR and/or a second MAT should systematically be performed, particularly when facing an acute clinical syndrome for which leptospirosis is suspected. As additional serological tools, newly developed ELISAs, both at the laboratory level and as rapid serological point-of-care tests, could also provide useful information, particularly when detecting IgM, which appears in the early stages of infection. These latest tests were unfortunately not available at the time of our study, but would represent an interesting prospect in similar future studies.

The MAT also has known limitations when it comes to precisely identifying the causative serogroup, mainly due to serologic cross-reactivity, which can result in positive titers for multiple serovars and even serogroups. This can be observed from our results (Supplementary Materials Table S1) and is well described in the literature [16,18,20]. The difficulty in drawing a conclusion from MAT results was obvious when considering the 14 cases from which *Leptospira* strains could be isolated, allowing us to test the consistency between the MAT and molecular typing. Out of those 14 cases, 9 were initially found to be clearly related to a specific serogroup when using the MAT (6 to Australis and 3 to Icterohaemorrhagiae), with 4 being “possibly” related to a certain serogroup (3 to Australis and 1 to Icterohaemorrhagiae) and 1 remaining fully inconclusive (Table 8). Interestingly, molecular typing was able to confirm ten out of the fourteen MAT serological characterizations but did not confirm the results for three of the strains, which were either “very likely” or “possibly” classified (with one MAT-typed Australis strain actually being Icterohaemorrhagiae and two Icterohaemorrhagiae strains being Australis based on molecular typing). Molecular typing was also able to provide an answer regarding the one strain for which the MAT result was “inconclusive”, showing that it belonged to the Australis serogroup (Table 10).

The results of our study also emphasize the important role of PCR in the primary diagnosis of leptospirosis. Out of the 41 *Leptospira* infected cases, 7 were either MAT-inconclusive (MAT+/−) or -negative (MAT−) but were found to be positive based on their PCR results. Overall, PCR was able to detect infection in 21 dogs, compared with 34 cases that were detected using the MAT. In addition, it should be noted that, out of these 21 PCR-positive samples, 6 were found to be positive solely for blood and 11 solely for urine, and 4 dogs were PCR-positive for both blood and urine. This confirms the literature findings that advise performing PCR on both types of samples; this is because, in the early phases of the disease, *Leptospira* genetic material is essentially found in blood, and, in the later phases, it is likely to be found in urine, even if shedding is intermittent [9].

Some inconsistency was observed between the PCR and culture results, which can be explained for the PCR-positive/culture-negative results by the susceptibility of *Leptospira*, the difficulty of growing these bacteria, and the limited time during which live bacteria are present in blood and/or urine. In contrast, PCR-positive/culture-negative results can be explained by the low number of bacteria, when the concentration of genetic material in the samples is below the limit of detection.

This study also confirmed the epidemiological importance of the Australis serogroup, representing 11 out of the 14 isolated strains. This result is in line with recently published data [6,7,8]. Interestingly, based on the MAT results (BS1 and/or BS2 when available), the Australis serogroup was considered as being “very likely” or “likely” involved in 48.8% (20/41) of the positive cases, whereas 5 cases were likely related to the Icterohaemorrhagiae serogroup; 16 cases remained inconclusive (Supplementary Materials Table S1). When considering the molecular typing of the 14 isolated strains, it resulted in 25 cases (61%) considered to be Australis-related and 4 being Icterohaemorrhagiae-related, with 12 cases remaining inconclusive. These data show that the Australis serogroup was dominant in the selected dog population.

One of the main goals of this study was also to show that recently developed molecular tools, such as PFGE, cgMSLT, and VNTR, are able to provide precise information about serogroups involved in disease, which can be relevant for an etiological diagnosis when suspecting an infection and for epidemiological purposes. In particular, new molecular techniques are now major tools, as it was recently shown that they allow for identifying the causative serogroup from blood and urine without necessarily undergoing a culturing step [27].

High-resolution subtyping using CgMLST sequence types (cgST) provided useful information, showing high diversity among the isolated strains. The fact that a large panel of pathogenic and genetically different field strains from the Australis serogroup could be isolated is actually an interesting and major outcome of this study, as it constitutes a good basis for further vaccine development, corresponding to epidemiological needs. It allows for choosing distant strains—a vaccine strain on the one hand and a challenge strain on the other—which is a prerequisite of the European Pharmacopoeia for validating challenge studies from a regulatory point of view. To the best of our knowledge, this should allow for the selection of a field L. Australis strain of canine origin as a vaccine strain and a highly virulent strain of proven pathogenicity as a challenge strain for the first time, thereby establishing the best conditions for demonstrating vaccine efficacy.

## 5. Conclusions

This study confirms that *Leptospira* field strains can be successfully isolated by implementing strict clinical case selection, sampling procedures, and processing, despite these bacteria being susceptible to the outdoor environment and being difficult to grow. We again bring to light the limitations of the MAT, both as a diagnostic tool for acute leptospirosis in the early stages and for identifying serogroups involved in the disease. A combination of different testing methods should, therefore, be implemented at the clinical practice level when suspecting leptospirosis, with PCR playing an important role besides the MAT. Finally, we confirm the epidemiological importance of the Australis serogroup, which represented a high proportion of the isolated field strains. From a clinical perspective, infection by this particular serogroup was shown to cause severe clinical signs and a high mortality rate, accompanied by bacterial excretion in a significant number of cases, thus representing a zoonotic risk that should not be underestimated. From a vaccine development point of view, this study has allowed for the collection of several and genetically different serovar Australis pathogenic strains of canine origin.

## Figures and Tables

**Table 1:** Leptospiral antigen panel used for the microscopic agglutination test.

Genus *Leptospira*	**Genomic Species**	**Serogroup**	**Serovar**	**Strain**	**Abbreviation**
	*interrogans*	Icterohaemorrhagiae	Icterohaemorrhagiae	ENVN	ICT
	*interrogans*	Icterohaemorrhagiae	Copenhageni	M 20	COP
	*interrogans*	Australis	Muenchen	München C 90	MUN
	*interrogans*	Australis	Australis	Ballico	AUS
	*interrogans*	Australis	Bratislava	Jez Bratislava	BRAT
	*interrogans*	Autumnalis	Autumnalis	Akiyami A	AKI
	*kirschneri*	Autumnalis	Bim	1051	BIM
	*interrogans*	Canicola	Canicola	Hond Utrecht IV	CAN
	*kirschneri*	Grippotyphosa	Grippotyphosa	Moskva V	GRIP
	*kirschneri*	Grippotyphosa	Vanderhoedoni	Kipod 179	VAN
	*noguchii*	Panama	Panama	CZ 214 K	PAN
	*undesignated*	Panama	Mangus	TRVL/CAREC 137774	MAN
	*interrogans*	Pomona	Pomona	Pomona	POM
	*kirschneri*	Pomona	Mozdok	5621	MOZ
	*interrogans*	Pyrogenes	Pyrogenes	Salinem	PYR
	*borgpetersenii*	Sejroe	Sejroe	M84	SJ
	*interrogans*	Sejroe	Saxkoebing	Mus 24	SAX
	*interrogans*	Sejroe	Wolffi	3705	WOLF
	*interrogans*	Sejroe	Hardjo	Hardjoprajitno	HJ
	*kirschneri*	Cynopteri	Cynopteri	3522C	CYN

**Table 3:** Consistency between MAT and PCR results.

		MAT											
		Positive			Doubtful			Negative			Total		
PCR-positive	Blood	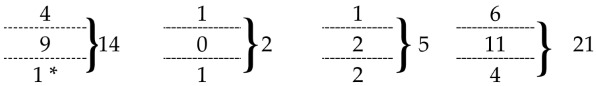											
	Urine												
	Blood + urine												
PCR-negative		20			5			18			43		
Total		34			7			23			64		

* Dog positive in urine and blood pool.

**Table 4:** Overall occurrence (N)/frequency (%) of the clinical syndromes at inclusion in the different dog populations.

Inclusion Criteria	All Dogs (n = 64)	Infected Dogs (n = 41)	Dogs with Negative Leptospirosis Status * (n = 18)
Acute renal failure	42 (65.6%)	32 (78.0%)	7 (38.9%)
Acute hepatitis	25 (39.1%)	15 (36.6%)	7 (38.9%)
Acute hemorrhagic gastroenteritis	16 (25.0%)	9 (21.9%)	6 (33.3%)
Glucosuria w/o hyperglycemia	13 (20.3%)	10 (24.4%)	1 (5.6%)
Hemorrhagic syndrome	2 (3.1%)	1 (2.4%)	1 (5.6%)

* Doubtful cases were excluded from the calculation.

**Table 5:** Clinical syndromes observed at inclusion in the 41 infected dogs.

With ARF at inclusion	ARF alone	14				14
	+AH	2	Without ARF at inclusion	AH	5	7
	+AHGE	3		AHGE	1	4
	+HS	1		Hs	0	1
	+GluU	7		GluU	0	7
	+AH + AHGE	2		AH + AHGE	3	5
	+AH + GluU	3		AH + GluU	0	3
	+AH + HS	0		AHGE + Hs	0	0
	Total “with ARF”	32		Total “without ARF”	9	41

ARF, acute renal failure; AH, acute hepatitis; AHGE, acute hemorrhagic gastroenteritis; HS, hemorrhagic syndrome; GluU, glucosuria without hyperglycemia.

**Table 6:** Clinical signs at inclusion for the 39 infected dogs from which clinical data were obtained.

Clinical Signs > 20%			Clinical Signs < 20%		
Lethargy	37	94.9%	Oligoanuria	6	15.4%
Anorexia	34	87.2%	Renal pain/hypertrophy	6	15.4%
Vomiting	25	64.1%	Congestion	5	12.8%
Dehydration	19	48.7%	Mucosal paleness	5	12.8%
Abdominal pain	15	38.5%	Hematochezia	4	10.3%
Diarrhea	14	35.9%	Ocular congestion	4	10.3%
Weight loss	11	28.2%	Dyspnea	3	7.7%
Icterus	11	28.2%	Melena	3	7.7%
Muscular weakness/pain	9	23.1%	Hepatomegaly	3	7.7%
PUPD	8	20.5%	Tremor	3	7.7%

Note: All other signs were observed in less than 7% of the dogs.

**Table 7:** Consistency between PCR and bacterial isolation depending on the biological material. Cases displaying discrepancies are shown in bold.

Dog ID	Culture/Isolation Results		PCR Results	
	Blood	Urine	Blood	Urine
A02	-	Pos	-	Pos
A07	-	Pos	-	Pos
A09	-	Pos	-	Pos
**B01**	**Pos**	-	-	-
**B02**	Pos	-	Pos	**Pos**
**B06**	**Pos**	-	Pos	**Pos**
F01	Pos	-	Pos *	
**H01**	-	**Pos**	-	-
**H03**	**Pos**	-	-	**Pos**
**Q01**	**Pos**	-	-	**Pos**
Q02	Pos	-	Pos	-
**S03**	**Pos**	Pos	-	Pos
T01	-	Pos	-	Pos
**U01**	-	**Pos**	**Pos**	-

* Positive in pooled blood/urine sample.

**Table 8:** Serological characterization based on MAT results for the 14 cases with bacterial isolation.

		ICTEROHAEMORRHAGIAE		CANICOLA	AUSTRALIS			GRIPPOTYPHOSA		POMONA		SEJROE				AUTOMNALIS		PANAMA		PYROGENES	CYNOPTERI	SEROLOGICAL CHARACTERIZATION BASED ON MAT
Serovar		ICT	COP	CAN	AUS	MUN	BRAT	GRIP	VAN	MOZ	POM	SJ	SAX	HJ	WOLF	AKI	BIM	PAN	MAN	PYR	CYN	
Case	BS #																					
A02	BS1	1280	1280	40	1280	2560	2560	1280	320	320	0	0	0	0	0	80	160	0	0	0	0	AUSTRALIS
	BS2	1280	1280	40	1280	2560	1280	640	0	160	0	0	0	0	0	160	160	0	0	0	0	
A07	BS1	320	1280	160	>5120	>5120	640	640	2560	640	160	0	0	0	0	160	2560	0	2560	80	0	Likely AUSTRALIS Possibly Grippo or Panama
	BS2	80	320	0	>5120	>5120	640	5120	5120	640	160	0	0	0	0	0	0	0	>5120	0	0	
A09	BS1	1280	5120	640	10,240	>10,240	10,240	320	320	0	0	80	0	320	160	0	0	0	1280	640	0	Likely AUSTRALIS (possibly Ictero)
	BS2	1280	5120	1280	640	1280	5120	0	80	0	0	0	0	0	0	0	0	0	160	1280	0	
B01	BS1	1280	2560	160	2560	1280	2560	0	0	0	0	0	0	0	0	1280	2560	0	0	40	0	INCONCLUSIVE (Australis/Ictero/Autumnalis)
	BS2	Dog died before second blood sampling																				
B02	BS1	5120	1280	640	0	0	0	0	0	0	0	0	0	0	0	0	0	0	0	0	0	ICTERO
	BS2	Dog died before second blood sampling																				
B06	BS1	1280	2560	0	0	320	160	0	0	0	0	0	0	0	0	0	0	0	0	0	0	ICTERO
	BS2	Dog died before second blood sampling																				
F01	BS1	1280	1280	80	320	320	640	0	0	80	80	80	0	0	0	0	0	0	0	80	0	ICTERO
	BS2	Dog died before second blood sampling																				
H01	BS1	160	640	80	0	0	0	0	0	0	0	0	0	0	0	0	0	0	0	640	0	AUSTRALIS
	BS2	400	800	400	6400	>12,800	>12,800	200	100	0	0	0	0	0	0	100	400	0	400	400	0	
H03	BS1	2560	10240	160	5120	20,480	20,480	0	0	0	0	0	0	0	0	160	160	80	640	0	0	AUSTRALIS
	BS2	Dog died before second blood sampling																				
Q01	BS1	0	200	100	0	0	0	0	0	0	0	0	0	0	0	0	0	0	0	0	0	AUSTRALIS
	BS2	800	800	100	6400	12,800	400	3200	3200	800	3200	0	0	0	0	3200	6400	0	12,800	800	12,800	
Q02	BS1	100	200	100	0	200	100	0	0	0	0	0	0	0	0	0	0	0	0	0	0	AUSTRALIS
	BS2	400	400	200	6400	12,800	6400	200	0	0	0	0	0	0	0	1600	800	0	800	100	0	
S03	BS1	3200	3200	0	100	200	200	200	100	0	100	3200	3200	200	0	0	200	200	400	400	800	Likely ICTERO or Sejroe
	BS2	Dog died before second blood sampling																				
T01	BS1	12,800	6400	100	12,800	12,800	800	800	800	0	200	0	0	0	0	1600	1600	200	6400	0	6400	AUSTRALIS
	BS2	1600	800	100	12,800	1600	800	1600	3200	800	200	0	0	0	0	200	6400	0	6400	0	6400	
U01	BS1	800	800	100	3200	12,800	3200	1600	3200	1600	6400	12,800	12,800	0	0	1600	12,800	100	12,800	200	12,800	Likely AUSTRALIS Possibly Grippo or Pomona
	BS2	400	400	100	6400	12,800	12,800	12,800	6400	12,800	1600	12,800	800	0	0	800	12,800	100	12,800	100	12,800	

Note: The values in the table are the MAT reciprocal dilution titers. The serovars against which the highest titers were observed for each case are displayed in red. Cases with questionable interpretation are highlighted with yellow/orange background.

**Table 9:** Leptospira isolate typing results of VNTR and PFGE and subtyping results of CgMLST.

Technique		RNA16S	cgMLST		VNTR, PFGE
Outcome		Species	Serogroup	Core Genome Sequence Typing (cgST)	Serovar
Dog Id	A02	*Leptospira interrogans*	Australis	cgST648	Bratislava
	A07		Australis	cgST648	Bratislava
	A09		Icterohaemorrhagiae	CgST167	Icterohaemorrhagiae/Copenhageni
	B01		Australis	cgST648	Bratislava
	B02		Australis	cgST648	Bratislava
	B06		Australis	cgST648	Bratislava
	F01		Icterohaemorrhagiae	CgST190	Icterohaemorrhagiae/Copenhageni
	H01		Australis	CgST613	Bratislava
	H03		Australis	CgST727	Bratislava
	Q01		Australis	CgST677	Bratislava
	Q02		Australis	CgST773	Bratislava
	S03		Icterohaemorrhagiae	CgST167	Icterohaemorrhagiae/Copenhageni
	T01		Australis	CgST648	Bratislava
	U01		Australis	CgST664-677	Bratislava

**Table 10:** Consistency between MAT serological characterization and molecular typing.

Case #	Suspected Serogroup (Based on MAT)	Confirmed Serogroup (Based on Molecular Typing)
A02	Australis	Australis
A07	Likely Australis Possibly Grippotyphosa or Panama	Australis
A09	Likely Australis (possibly Icterohaemorrhagiae)	Icterohaemorrhagiae
B01	INCONCLUSIVE (Australis or Icterohaemorrhagiae or Autumnalis)	Australis
B02	Icterohaemorrhagiae	Australis
B06	Icterohaemorrhagiae	Australis
F01	Icterohaemorrhagiae	Icterohaemorrhagiae
H01	Australis	Australis
H03	Australis	Australis
Q01	Australis	Australis
Q02	Australis	Australis
S03	Likely Icterohaemorrhagiae or Sejroe	Icterohaemorrhagiae
T01	Australis	Australis
U01	Likely Australis Possibly Grippotyphosa or Pomona	Australis

**Table 11:** Main syndromes at inclusion of the 14 isolation-positive cases.

Clinical Syndrome	Serogroup		Total
	Australis (n = 11)	Icterohaemorrhagiae (n = 3)	
Acute renal failure	11	3	14 (100%)
Acute hepatitis	2	2	4 (28.6%)
Acute hemorrhagic gastroenteritis	2	0	2 (14.3%)
Hemorrhagic syndrome	0	0	0
Glucosuria	4	1	5 (35.7%)
Survival	7	1	8 (57.1%)

## Data Availability

The original contributions presented in the study are included in the article/Supplementary Material, further inquiries can be directed to the corresponding author.

## Supplementary Materials

The following supporting information can be downloaded at https://www.mdpi.com/article/10.3390/microorganisms12101946/s1, Table S1: MAT and PCR results of the 41 positive cases, with molecular typing of the 14 isolated strains.
