# Supplementary material for: Isolation of Virulent Leptospira Serogroup Australis Field Strains from Symptomatic Dogs for Canine Leptospiral Vaccine Development
Source: Microorganisms. 2024 Sep 25;12(10):1946. doi: 10.3390/microorganisms12101946 (PMC11509459; doi:10.3390/microorganisms12101946)
Supplement: Supplementary file 1 [file microorganisms-3170205-supplementary.pdf › microorganisms-3170205-supplementary.pdf]

Serogroup -->		SG ICT		SG CAN	SG AUS			SG GRIP		SG POM		SG SEJ				SG AUT		SG PAN		SG PYR	SG CYN		SG ICT		SG CAN	SG AUS			SG GRIP		SG POM		SG SEJ				SG AUT		SG PAN		SG PYR	SG CYN					
	MAT (Blood Sample #1)																					MAT (Blood Sample #2)																						Interpretation			
Case number	Blood sampling date #1	IH	COP	CAN	AUS	MUN	BRAT	GRIP	VAN	MOZ	POM	SJ	SAX	HJ	WOLF	AKI	BIM	PAN	MAN	PYR	CYN	Blood sampling date #2	IH	COP	CAN	AUS	MUN	BRAT	GRIP	VAN	MOZ	POM	SJ	SAX	HJ	WOLF	AKI	BIM	PAN	MAN	PYR	CYN	PCR result	Interpret. BS1	Interpr. BS1/BS2	Mol. Typing	Overall Interpret.
A02	07/08/10	1280	1280	40	1280	2560	2560	1280	320	320	0	0	0	0	0	80	160	0	0	0	0	25/08/10	1280	1280	40	1280	2560	1280	640	0	160	0	0	0	0	0	160	160	0	0	0	0	+	AUS	AUS	AUS	AUS
A04	14/09/10	320	1280	640	320	1280	320	640	640	160	640	0	0	0	0	160	640	40	160	0	0	28/09/10	320	1280	1280	160	1280	320	640	1280	160	1280	0	0	0	0	160	640	0	640	0	0	-	AUS/ICT?	Inconcl.		Inconcl.
A05	04/10/10	160	320	160	1280	1280	640	0	0	0	0	0	0	0	0	0	0	80	160	0	0	19/10/10	160	160	160	1280	1280	320	0	0	0	0	0	0	0	0	0	0	80	320	0	0	-	AUS	AUS		AUS
A07	11/10/11	320	1280	160	>5120	>5120	640	640	2560	640	160	0	0	0	0	160	2560	0	2560	80	0	03/11/11	80	320	0	>5120	>5120	640	5120	5120	640	160	0	0	0	0	0	0	0	>5120	0	0	+	AUS	AUS	AUS	AUS
A09	13/12/11	1280	5120	640	10240	>10240	10240	320	320	0	0	80	0	320	160	0	0	0	1280	640	0	02/02/12	1280	5120	1280	640	1280	5120	0	80	0	0	0	0	0	0	0	0	0	160	1280	0	+	AUS	Inconcl.	ICT	ICT
B01	03/03/11	1280	2560	160	2560	1280	2560	0	0	0	0	0	0	0	0	1280	2560	0	0	40	0	Died	NA	NA	NA	NA	NA	NA	NA	NA	NA	NA	NA	NA	NA	NA	NA	NA	NA	NA	NA	NA	-	Inconcl.	X	AUS	AUS
B02	24/03/11	5120	1280	640	0	0	0	0	0	0	0	0	0	0	0	0	0	0	0	0	0	Died	NA	NA	NA	NA	NA	NA	NA	NA	NA	NA	NA	NA	NA	NA	NA	NA	NA	NA	NA	NA	+	ICT	X	AUS	AUS
B04	28/05/11	1280	1280	80	>5120	>5120	1280	80	80	160	80	0	0	0	0	80	80	160	2560	0	0	Lost	NA	NA	NA	NA	NA	NA	NA	NA	NA	NA	NA	NA	NA	NA	NA	NA	NA	NA	NA	NA	-	AUS	X		AUS
B06	09/06/11	1280	2560	0	0	320	160	0	0	0	0	0	0	0	0	0	0	0	0	0	0	Died	NA	NA	NA	NA	NA	NA	NA	NA	NA	NA	NA	NA	NA	NA	NA	NA	NA	NA	NA	NA	+	ICT	X	AUS	AUS
D01	19/10/10	40	0	40	0	0	0	0	0	0	0	0	0	0	0	0	0	0	0	40	0	Died	NA	NA	NA	NA	NA	NA	NA	NA	NA	NA	NA	NA	NA	NA	NA	NA	NA	NA	NA	NA	+	Inconcl.	X	X	Inconcl.
D02	04/04/11	1280	2560	80	1280	10240	10240	0	0	0	0	0	0	0	0	320	1280	40	640	80	0	Lost	NA	NA	NA	NA	NA	NA	NA	NA	NA	NA	NA	NA	NA	NA	NA	NA	NA	NA	NA	NA	-	AUS	X		AUS
E01	12/10/10	1280	1280	320	2560	>5120	>5120	0	0	0	0	0	0	0	0	2560	0	80	320	0	0	Died	NA	NA	NA	NA	NA	NA	NA	NA	NA	NA	NA	NA	NA	NA	NA	NA	NA	NA	NA	NA	-	AUS	X		AUS
E03	08/12/10	0	0	160	0	0	0	0	0	0	0	0	0	0	0	0	0	0	0	0	0	27/12/10	0	0	160	0	0	0	0	0	0	0	0	0	0	0	0	0	0	0	0	0	+	Inconcl.	Inconcl.	X	Inconcl.
E04	29/01/11	320	1280	0	0	0	0	0	0	0	0	0	0	0	0	0	0	0	0	0	0	14/02/11	320	1280	0	0	0	0	0	0	0	0	0	0	0	0	0	0	0	0	0	0	+	ICT	ICT	X	ICT
E05	17/02/11	80	80	80	640	160	160	0	0	0	0	0	0	0	0	0	0	0	0	0	0	Lost	NA	NA	NA	NA	NA	NA	NA	NA	NA	NA	NA	NA	NA	NA	NA	NA	NA	NA	NA	NA	-	AUS?	X		Inconcl.
F01	22/12/10	1280	1280	80	320	320	640	0	0	80	80	80	0	0	0	0	0	0	0	80	0	Died	NA	NA	NA	NA	NA	NA	NA	NA	NA	NA	NA	NA	NA	NA	NA	NA	NA	NA	NA	NA	+	ICT	X	ICT	ICT
G01	11/02/11	160	160	80	0	0	0	0	0	0	0	0	0	0	0	0	0	0	0	0	0	Died	NA	NA	NA	NA	NA	NA	NA	NA	NA	NA	NA	NA	NA	NA	NA	NA	NA	NA	NA	NA	+	ICT?	X	X	Inconcl.
G03	12/05/11	640	1280	0	5120	2560	1280	0	0	0	0	0	0	0	0	0	0	0	0	0	0	Died	NA	NA	NA	NA	NA	NA	NA	NA	NA	NA	NA	NA	NA	NA	NA	NA	NA	NA	NA	NA	-	AUS	X		AUS
G04	08/08/11	80	40	160	10240	2560	80	80	80	0	0	0	0	0	0	160	160	0	2560	0	0	23/08/11	0	0	100	3200	1600	100	0	0	0	0	0	0	0	0	0	0	0	800	0	0	-	AUS			AUS
G05	26/10/11	640	1280	640	0	0	0	80	0	0	0	0	0	0	0	40	80	0	0	1280	0	10/11/11	400	400	400	0	0	0	0	0	0	0	0	0	0	0	0	0	0	0	400	0	-	Inconcl.	Inconcl.		Inconcl.
H01	04/08/10	160	640	80	0	0	0	0	0	0	0	0	0	0	0	0	0	0	0	640	0	18/08/10	400	800	400	6400	>12800	>12800	200	100	0	0	0	0	0	0	100	400	0	400	400	0	-	Inconcl.	AUS	AUS	AUS
H02	23/08/10	1280	1280	0	2560	2560	2560	0	0	0	0	320	0	0	0	0	0	0	0	0	0	Died	NA	NA	NA	NA	NA	NA	NA	NA	NA	NA	NA	NA	NA	NA	NA	NA	NA	NA	NA	NA	+	AUS	X	X	AUS
H03	22/03/11	2560	10240	160	5120	20480	20480	0	0	0	0	0	0	0	0	160	160	80	640	0	0	Died	NA	NA	NA	NA	NA	NA	NA	NA	NA	NA	NA	NA	NA	NA	NA	NA	NA	NA	NA	NA	+	AUS	X	AUS	AUS
J02	05/07/11	>5120	>5120	320	>5120	>5120	>5120	2560	2560	640	2560	0	0	0	0	1280	>5120	160	>5120	160	0	Died	NA	NA	NA	NA	NA	NA	NA	NA	NA	NA	NA	NA	NA	NA	NA	NA	NA	NA	NA	NA	-	Inconcl.	X		Inconcl.
J03	19/09/11	0	0	0	640	1280	320	0	0	0	0	80	160	40	0	0	0	0	0	0	0	05/10/11	0	0	0	320	1280	160	0	0	0	0	80	640	0	0	0	0	0	0	0	0		AUS	AUS	X	AUS
J05	26/10/11	40	40	0	1280	320	40	0	0	0	0	160	1280	80	80	0	0	0	80	0	0	Lost	NA	NA	NA	NA	NA	NA	NA	NA	NA	NA	NA	NA	NA	NA	NA	NA	NA	NA	NA	NA	-	Inconcl.	X		Inconcl.
L01	04/10/11	160	160	320	0	0	0	0	0	0	0	0	0	0	0	0	0	0	80	0	0	Died	NA	NA	NA	NA	NA	NA	NA	NA	NA	NA	NA	NA	NA	NA	NA	NA	NA	NA	NA	NA	+	Inconcl.	X	X	Inconcl.
M01	07/06/11	5120	1280	80	10240	10240	1280	0	0	0	0	0	0	0	0	0	0	0	40	160	0	Died	NA	NA	NA	NA	NA	NA	NA	NA	NA	NA	NA	NA	NA	NA	NA	NA	NA	NA	NA	NA	+	AUS	X		AUS
Q01	30/05/13	0	200	100	0	0	0	0	0	0	0	0	0	0	0	0	0	0	0	0	0	N/A	800	800	100	6400	12800	400	3200	3200	800	3200	0	0	0	0	3200	6400	0	12800	800	12800	+	NEG	Inconcl.	AUS	AUS
Q02	22/09/14	100	200	100	0	200	100	0	0	0	0	0	0	0	0	0	0	0	0	0	0	N/A	400	400	200	6400	12800	6400	200	0	0	0	0	0	0	0	1600	800	0	800	100	0	+	NEG	AUS	AUS	AUS
R01	04/03/14	0	0	200	0	0	100	0	0	0	0	0	1600	200	0	0	100	0	0	0	0	24/03/14	0	0	100	0	0	0	0	0	0	0	0	1600	0	0	0	0	0	0	0	0	-	SAX	SAX		SAX
S01	04/09/13	0	0	0	3200	3200	400	3200	100	0	100	0	400	0	0	0	200	0	800	0	400	Died	NA	NA	NA	NA	NA	NA	NA	NA	NA	NA	NA	NA	NA	NA	NA	NA	NA	NA	NA	NA	-	Inconcl.	X		Inconcl.
S03	30/10/14	3200	3200	0	100	200	200	200	100	0	100	3200	3200	200	0	0	200	200	400	400	800	Died	NA	NA	NA	NA	NA	NA	NA	NA	NA	NA	NA	NA	NA	NA	NA	NA	NA	NA	NA	NA	+	Inconcl.	X	ICT	ICT
T01	01/07/13	12800	6400	100	12800	12800	800	800	800	0	200	0	0	0	0	1600	1600	200	6400	0	6400	15/07/13	1600	800	100	12800	1600	800	1600	3200	800	200	0	0	0	0	200	6400	0	6400	0	6400	+	ICT/AUS	Inconcl.	AUS	AUS
T02	08/08/14	1600	1600	100	400	400	3200	0	0	400	0	0	200	0	0	0	200	400	400	800	0	Died	NA	NA	NA	NA	NA	NA	NA	NA	NA	NA	NA	NA	NA	NA	NA	NA	NA	NA	NA	NA	-	AUS	X		AUS?
U01	24/09/13	800	800	100	3200	12800	3200	1600	3200	1600	6400	12800	12800	0	0	1600	12800	100	12800	200	12800	15/01/14	400	400	100	6400	12800	12800	12800	6400	12800	1600	12800	800	0	0	800	12800	100	12800	100	12800	+	Inconcl.	Inconcl.	AUS	AUS
U02	22/11/13	800	400	100	0	0	400	0	100	400	0	0	400	0	0	0	0	0	0	0	0	Lost	NA	NA	NA	NA	NA	NA	NA	NA	NA	NA	NA	NA	NA	NA	NA	NA	NA	NA	NA	NA	+	Inconcl.	X		Inconcl.
W01	10/09/13	0	0	0	200	800	200	0	0	0	0	0	0	0	0	0	0	0	0	0	0	Died	NA	NA	NA	NA	NA	NA	NA	NA	NA	NA	NA	NA	NA	NA	NA	NA	NA	NA	NA	NA	-	AUS	X		AUS
W02	10/10/13	0	0	0	400	400	400	0	0	0	0	0	0	0	0	0	0	0	0	0	0	30/10/13	0	0	0	200	800	800	0	0	0	0	0	0	0	0	0	0	0	0	0	0	-	Aus?	AUS		AUS
W03	29/11/13	0	0	0	800	400	1600	0	0	0	0	0	0	0	0	0	0	0	200	0	0	16/12/13	0	0	0	800	1600	1600	0	0	0	0	0	0	0	0	0	0	0	200	0	0	+	AUS	AUS		AUS
W04	19/11/13	100	0	0	1600	800	1600	0	0	0	0	400	0	400	0	0	100	100	800	0	100	Died	NA	NA	NA	NA	NA	NA	NA	NA	NA	NA	NA	NA	NA	NA	NA	NA	NA	NA	NA	NA	-	AUS	X		AUS
41	41	41	41	41	41	41	41	41	41	41	41	41	41	41	41	41	41	41	41	41	41	18	41	41	41	41	41	41	41	41	41	41	41	41	41	41	41	41	41	41	41	41	21			14	

Table S1: MAT and PCR results of the 41 positive cases, with molecular typing results of the 14 isolated strains.

Titers are expressed as reverse dilutions. For easier reading, color intensities from pale yellow to purple reflect increase seropositivity. In the last columns, cases negative for bacterial isolation and positive for PCR appear in yellow, cases positive for bacterial isolation and negative for PCR appear in blue, and cases positive for both PCR and bacterial isolation appear in green. BS1= blood sampling 1, BS2 = blood sampling 2.

Based only on the MAT results (BS1 and/or BS2 when available), the Australis serogroup was considered as being “very likely” or “likely” involved in 48.8% (20/41) of the positive cases, whereas 5 cases were likely related to the Icterohaemorrhagiae serogroup; 16 cases remained inconclusive. Based on the combined MAT/molecular typing results (“overall interpretation”), serogroup Australis was confirmed/likely to be involved in 61.0% (25/41) of the cases and serogroup Icterohaemorrhagiae in 9.8% (4/41) of the cases. 11 cases remained inconclusive.
